# A Scoping Review of the Evidence regarding Assessment and Management of Psychological Features of Shoulder Pain

**DOI:** 10.1155/2021/7211201

**Published:** 2021-09-30

**Authors:** Maryam Farzad, Joy C. MacDermid, David C. Ring, Erfan Shafiee

**Affiliations:** ^1^School of Physical Therapy, Department of Health and Rehabilitation Sciences, University of Western Ontario, London, Ontario, Canada; ^2^Roth McFarlane Hand and Upper Limb Centre, St. Joseph's Hospital, London, Ontario, Canada; ^3^Department of Occupational Therapy, University of Social Welfare and Rehabilitation Sciences, Tehran, Iran; ^4^Rehabilitation Science McMaster University, Hamilton, ON, Canada; ^5^Surgery and Psychiatry, Dell Medical School, The University of Texas at Austin, Texas, USA

## Abstract

**Methods:**

A scoping review of research studies identified through PubMed, EMBASE, and CINAHL and graduate theses identified using Google Scholar was conducted to determine studies and systematic reviews that addressed the management of psychological aspects of shoulder pain with or without neck pain. The search terms included psychological factors, anxiety, depression, catastrophic thinking, fear of movement, and psychological treatments. Two investigators screened study titles and abstracts. Data extraction, content analysis, and thematic coding focused on the dimensions of pain addressed (emotional, behavioural, and cognitive) and treatment approaches used (dimensions targeted, specific treatment parameters) and the linkage between treatment targets/rationale with interventions/outcomes measured.

**Results:**

Ten studies (seven randomized trials and three cohorts) were identified that addressed the psychological aspects of shoulder pain. Out of seven RCTs, four compared psychological interventions with usual care. Eight studies used cognitive approaches, including emotional freedom techniques (EFT), pain coping strategies (PCS), physical-cognitive-mindfulness training (PCMT), psychological flexibility, face-to-face cognitive-behavioural treatment (CBT), and cognitive therapy using virtual reality (V.R.). Three studies used the behavioural approaches as their intervention, including behavioural therapy and Graded Exercise Therapy (GET). Pain intensity was addressed as the primary outcome in two studies and as a secondary outcome in five studies. Cognitive factors were evaluated in 50% of the articles using nine different measures. Emotional factors were evaluated in 80% of articles using ten different measures. Reduction of pain intensity and catastrophic thinking concerning pain was achieved in most studies using a biopsychosocial approach (70%). Applying a behavioural approach was associated with reductions in kinesiophobia and pain catastrophizing. Cognitive approaches had a positive association with reductions in the emotional aspect of pain. Only one study specifically linked rationale or specific physical and psychosocial treatment targets with the treatments provided and outcomes measured.

**Conclusions:**

Small pools of studies indicate that the rationale and treatment targeting are poorly defined in biopsychosocial interventions for shoulder pain. However, these benefits have been demonstrated when cognitive or behavioural components are added to the standard physical treatment of shoulder pain. A better definition of treatment targets, description of intervention components, and linkage of outcomes to targets are needed to advance our understanding of optimizing bio-psychosocial approaches.

## 1. Introduction

Musculoskeletal (MSK) disorders affecting the hand and upper extremity are common [1] and can result in persistent pain. Patients that seek care for daily pain are often referred for therapy with the primary goal of limiting illness, improving function, and potentially improving pain self-management.

The considerable variation in pain intensity across patients with a similar injury and presumed similar nociceptive input is partially explained by mental and social factors [2]. Despite mounting evidence of the importance of psychological factors such as cognitive coping strategies (catastrophic thinking, fear of movement) and psychological distress (symptoms of depression and anxiety) as mediators of pain intensity for given nociception [3], the integration of these constructs into evaluation and treatment of upper extremity MSK conditions is limited.

Shoulder pain is second only to low back pain (LBP) among costs associated with the care of musculoskeletal disorders [4]. The cognitive-behavioural model of pain acknowledges the importance of underlying tissue pathology as a source of pain and highlights the critical role of psychological factors in pain [5]. Cognitive factors (such as self-efficacy and perceived helplessness), emotional factors (such as anxiety and depression), and behavioural factors have influenced pain perception and how one adapts to pain [5, 6]. Recent studies showed that pain perception in patients with persistent pain is influenced by pain emotion and cognition, which are considered psychological aspects of pain [7].

Although psychosocial approaches have been increasingly studied for people with LBP [8], the extent to which this has been integrated into the clinical management of chronic shoulder pain is limited. Understanding how psychological factors have been considered in the assessment, treatment targeting, and outcome evaluation would provide a start point for a more explicit discussion of how these factors could be consistently defined, studied, and implemented within rehabilitation programs.

The primary purpose of this scoping review was to identify and map the state of the literature for studies that integrate psychological management into the treatment of shoulder pain and classify the behavioural, cognitive, and emotional dimensions of pain that are considered in the evaluation as treatment targets. The secondary purpose was to map the evidence and describe the potential impacts of psychological interventions on pain and pain-related psychological factors.

## 2. Method

According to accepted methodology, a scoping review was conducted: identifying the research question, identifying the relevant studies, selecting the studies, charting the data (data extraction), collating, summarizing, and reporting the results [9, 10]. All relevant studies that met inclusion/exclusion criteria, regardless of quality, were included [11].

### 2.1. Identification and Selection of Studies

Relevant full-text peer-reviewed articles were identified in a search of the following databases: PubMed, EMBASE, CINAHL, and PsycINFO and Google Scholar. Grey literature was searched on Google. All years until April 2021 were searched with the following combinations of keyword: chronic pain, evaluation, shoulder, upper extremity, psychological factors, anxiety, depression, catastrophizing factor, fear of movement, and cognitive-behavioural treatment. These keywords were expanded using MESH terms and synonym searches (((((((((Chronic Shoulder Pain [MeSH Terms]) AND (Psychological factors[MeSH Terms])) OR (depression[MeSH Terms])) OR (anxiety[MeSH Terms])) OR (Catastrophizing[MeSH Terms])) OR (fear of movement[MeSH Terms])) AND (Treatment[MeSH Terms])) OR (evaluation[MeSH Terms])) OR (outcome[MeSH Terms])).

### 2.2. Study Selection

We included full-text original clinical research in English language papers that used any psychological intervention alone or along with usual cares for pain in patients with shoulder, upper extremity, neck, or upper back regions. We included studies in which more than 30% of patients had shoulder/neck pain. Forward and backward citation tracing was used, and the reference lists of the studies were hand-searched to check for additional studies. No restrictions were placed on the date of the study search to ensure inclusion of the full breadth of the literature. The exclusion criteria included papers that did not contain primary quantitative data on psychological factors in patients with shoulder pain, including qualitative studies, systematic reviews, and editorial pieces. Conference presentations, thesis or dissertations, editorial guidelines, and reviews were excluded.

**Figure 1:**
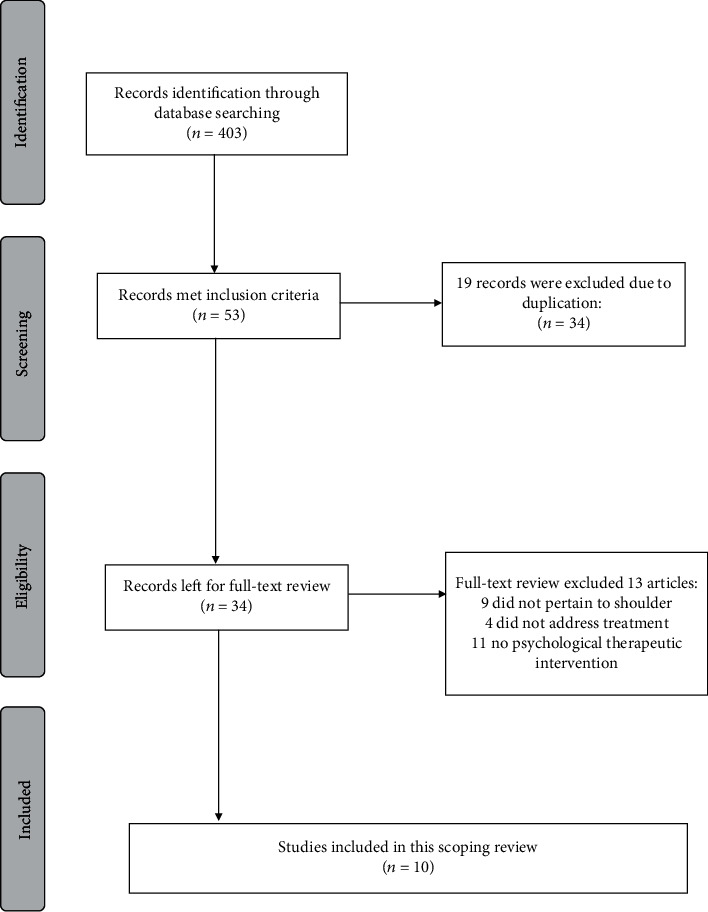
PRISMA flow chart of the selection of the studies for inclusion in the review.

Titles and abstracts of articles were independently reviewed by two authors to apply inclusion/exclusion criteria and delete duplications. Full texts of potentially relevant articles were retrieved and scrutinized by two of authors (M.F.) and (E.S.) for consensus before final inclusion in the study. In case of disagreement, a third party reviewer would be consulted (J.M.). We excluded the studies that failed to meet any one of the inclusion criteria. This review adhered to the Preferred Reporting Items for Reviews (PRISMA) criteria, and a flow diagram (Figure 1) of the included studies is provided. However, we did not register this protocol since scoping reviews are currently ineligible for registration.

### 2.3. Charting the Data

A total of 10 articles were included in this review (Figure 1). A study-specific data extraction sheet was devised. The number of participants, study methods, interventions, and outcome measures used to assess pain and psychological aspects of pain were extracted and organized in to table format (Table 1). This process was double-checked with another researcher. An additional table was created to summarize the characteristic of the psychological interventions, the study's main aim, and used outcome measures (Table 2). Charting was done iteratively and updated and refined if needed based on the results from summarizing.

### 2.4. Collating, Summarizing, and Reporting the Results

Consistent with scoping review methodology, we described the extent and nature of the available evidence, without considering the quality. We use descriptive synthesis to present the results to provide a wide range of details and diverse integrated evidence. Results were summarized according to the type of psychological intervention, the methods of evaluating psychological traits, and the outcomes on reduction pain or pain-related factors. The psychological factors were categorized as cognitive, emotional, and behavioural factors.

To make sense of the extracted data, we used a thematic analysis to gather information and identify all themes related to psychological factors related to pain. Inductive analysis was used to (1) extract meanings (codes) related to psychological factors in interventions, evaluations, and outcomes; (2) grouping the codes based on their similarity; and (3) categorize and summarizing the extracted meanings into three psychological categories related to pain perception: emotional, behavioural, and cognitive. Two of the reviewers (M.F. and E.S.) conducted coding, and it was checked by the third reviewer in case of disagreement (J.M.). We added a map to visually represent the findings in a broader context to further clinical practice, research, and policy.

## 3. Results

### 3.1. Search Results

Among the 403 studies identified by the search strategy, 34 studies met the inclusion and exclusion criteria and passed the abstract and title review. During the full-text review, nine studies were excluded because they did not pertain to shoulder [5, 12–19], four studies did not address treatment [20–23], and 11 studies did not use psychological interventions, leaving ten studies in total. We decided to keep one study in which 30% of the patients had shoulder or neck pain, and others had general pain or back pain due to its design and sample size, matching the psychological profile with treatment (Figure 1).

The characteristics of the study design are in Table 1. Among the ten remaining studies (Figure 1 and Table 1), there were seven randomized controlled trials (RCTs) [22, 24–27] and three cohort studies. The follow-up evaluation times ranged from 1 to 12 months. The outcomes evaluated included pain and psychological aspects of the pain experience.

### 3.2. Conceptualization and Themes

**Figure 2:**
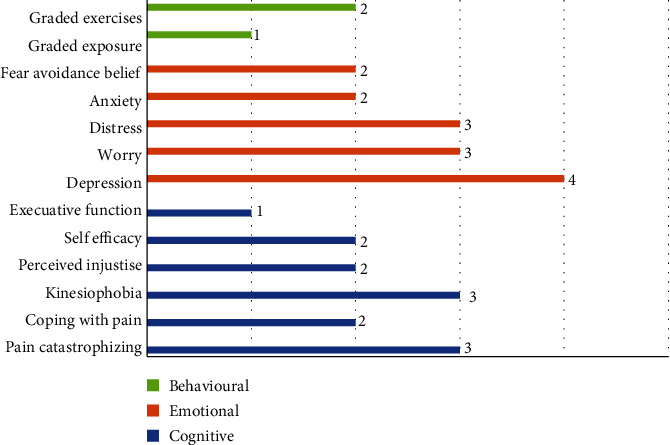
Frequency of the extracted construct from the included papers.

Eight constructs represented in the literature were classified as cognitive factors, including pain self-efficacy [27], pain catastrophizing [28, 29], perceived injustice (internal and external locus Of control) [26, 28], attention [29] executive functioning, memory [30], psychological inflexibility [18], kinesiophobia [24, 29, 31], and coping strategies [18, 27, 28] (Figure 2).

Seven constructs classified as emotional factors were extracted from the literature, including depression [18, 24–26, 30], distress [25], anxiety [24, 26, 29] and health anxiety [18], fear of avoidance belief [24, 28] and negative affectivity [24, 28, 29, 32], and pain-specific worries [24]. Graded exercise [24, 28, 29, 32] and exposure are categorized as behavioural factors (Figure 2).

### 3.3. Intervention Types

**Figure 3:**
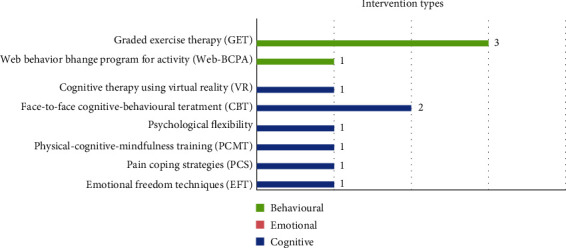
Frequency of the intervention types based on the psychological aspects of pain.

Seven studies included cognitive approaches, four behavioural, and none used emotional approaches as foundations of their treatment plan (Figure 3). Of the seven articles (70%) using cognitive approaches, the following specific types of treatment were implemented: emotional freedom techniques (EFT) [25], pain coping strategies (PCS) [33], physical-cognitive-mindfulness training (PCMT) [32], psychological flexibility [18], face-to-face cognitive-behavioural treatment (CBT) [24, 26], and cognitive therapy using virtual reality (V.R.) [30].

EFT is an exposure therapy that combines two other techniques: exposure therapy and cognitive therapy, with acupoint stimulation in pressure or percussion with the fingertips [25]. PCS [33] uses active (attempts to control the pain or to act despite the pain) and passive coping strategies (withdrawing from activities due to pain) to overcome pain. PCMT [32] consists of slow joint mobility exercises, different strength training exercises with elastic bands, and cognitive behavioural therapy in which education and counselling about the fear of movement occur. Psychological flexibility [18] is a cognitive intervention to promote acceptance, mindfulness, values-based action, and cognitive diffusion. V.R., with Duo Rehabilitation System, was used in one study as an experimental robotic platform that provides cognitive and affective relief and improves function [30]. Duo Rehabilitation System is a robotic table with computerized forearm support, a display, a laptop for the therapist station, and a remote clinical server that includes a variety of rehabilitation games which supports behavioural change in activity. Patients are encouraged to play nine games with the aim of motor (shoulder, elbow, and grasp), emotive, and cognitive (executive function focusing, short-term and delayed memory, working memory, and task sequencing training). This system measures when patients can continue to complete the activities, to encourage increased activity despite the pain. Cognitive-behavioural treatment (CBT) was used in three studies as a cognitive approach to identify and mitigate pain-related psychological factors, including negative cognitive, behavioural, and emotional factors [24, 26]. One study used both behavioural therapy and CBT based on evaluating a patient's psychological profile, and they matched the treatment approach to their evaluation [24]. CBT addresses maladaptive thinking, leading to maladaptive emotional and behavioural responses, and encourages adaptive thinking, which supports more positive emotions and behavioral responses.

Four studies that included the web behaviour change program for activity (Web-BCPA) in [27] and Graded Exercise Therapy (GET) [24, 28, 29] were classified as behavioural approaches, but also included cognitive elements. All implement a graded activity program where interventions are scheduled and revised based on the patient's physical status and tolerance with the start point based on their initial activity level ability. In a graded activity program, activities are used as treatments to increase the level of activity daily living. The selection and progression of meaningful daily activities are based on enhancing patient tolerance and provide carryover to incased function in daily life.

### 3.4. Measurements

Pain intensity was considered a primary outcome measure in two studies [27, 30]. Five studies evaluated pain intensity as their secondary outcome measure [19, 24–26, 32].

Six cognitive factors were evaluated in 50% of the articles using nine different measures. Pain catastrophizing was evaluated in three studies, using the Pain Catastrophizing Scale (PCS) [24, 28] [29]. Coping with pain was evaluated in two studies using PCS subscales [28] and Pain Coping Questionnaire (PCQ) [27]. Kinesiophobia was evaluated in three studies using the Tampa Scale of Kinesiophobia (TSK) [24, 29, 31]. Perceived injustice and locus of control were evaluated in two studies, using the Pain Coping and Cognition List (PCCL) [28] and Attention Module and Multidimensional Health Locus of Control questionnaire (Form A) (MHLC) [26]. One study evaluated self-efficacy using General Self-Efficacy Scale (GSE) and Arthritis Self-Efficacy Scale (ASES) [27] (Table 2).

**Figure 4:**
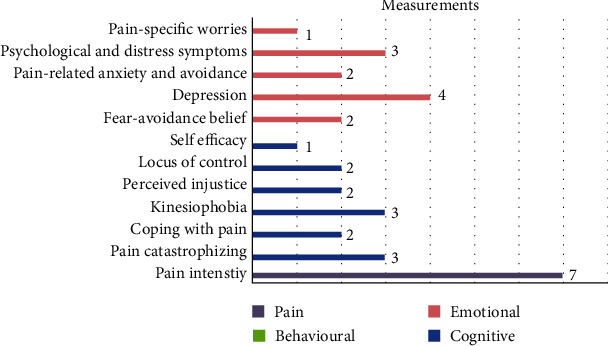
Frequency of the measurements area based on psychological aspect of pain.

Six emotional factors were evaluated in 80% of the articles, using ten different measures. Different aspects of emotion were considered, including depressive symptoms, psychological symptoms and distress, pain-related anxiety and avoidance, pain-specific worries, and fear-avoidance belief. These were primary or secondary targets of the psychological interventions [18, 24–26, 30, 33]. Fear-avoidance belief was evaluated in two studies using the Fear Avoidance belief questionnaire (FABQ) [24, 28]. Depression was evaluated in four studies using the Hospital Anxiety and Depression Scale (HADS_d, A) [24, 29], British Columbia Major Depression Inventory [18], and Beck Depression Inventory [30]. Pain-related anxiety and avoidance were evaluated in two studies using the Pain Anxiety Symptom [18] and Spielberg State-Trait Anxiety Scale (STAI I-II) [26]. Hopkins Symptom Checklist (HSCL) [26] and Symptom assessment-45 [24, 25] were used to evaluate psychological and distress symptoms in three studies. Pain-specific worries were evaluated in one study using Global Worry Testing [24] (Table 2 and Figure 4).

### 3.5. Effect of Interventions on Pain

GET, exercises with a behavioural approach, CBT, Coping Strategies, Psychological Flexibility, and V.R. had positive effects on pain reduction compared to usual care (Table 2) [18, 26, 27, 29, 30, 33]. Web-BCPA, EFT, matching therapy to psychological features, and physical cognitive-mindfulness did not affect reducing pain intensity.

### 3.6. Effect of Interventions on Pain-Related Psychological Factors

Two aspects of cognitive factors were improved by using psychological interventions. Pain catastrophizing was improved by using the Web-BCPA [27], GET, and CBT [24, 28, 29]. In one of the studies, CBT or GET was used to match the patient's psychological profile. Kinesiophobia was improved by using GET in one study [29].

Five aspects of emotional factors were improved using different interventions; fear avoidance, depression and anxiety, worry, and depressive symptom. Fear-avoidance belief was improved by using either CBT or GET based on matching with patient's psychological profile [24] and PCMT [32]. Matching treatment to psychological profile using either CBT or GET [24], EFT [25], and BrightArm Duo therapy [30] had a positive effect on depression, anxiety, and worry improvement. Depressive symptoms were improved using CBT [26] and P.F. [18] (Table 2).

### 3.7. The Rational Link between Intervention and Outcomes

**Figure 5:**
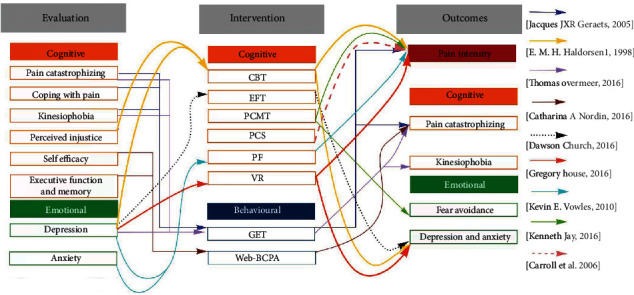
Mapping of the available evidence on usage of the psychological factors in evaluation and treatment of the patients with persistent shoulder and neck pain. Each arrow indicates each study and shows the psychological aspects they targeted in their evaluation, treatment, and outcomes.

One study linked treatment approaches, evaluation, and outcomes [24]. Their method had a positive effect on reducing pain intensity, pain catastrophizing, fear-avoidance belief, anxiety, depression, and worry about pain. They also evaluated both cognitive and emotional aspects of pain (Figure 5).

## 4. Discussion and Conclusion

### 4.1. Discussion

This scoping review identified ten studies, including seven RCTs and three cohorts, that included psychological factors in designing the treatment plans and evaluation for patients with persistent shoulder pain. This study represents a mapping of the evidence for psychological factors related to pain in the evaluation, intervention, and outcomes of the patients with persistent shoulder pain. We identified eleven themes classified into three psychological categories related to perception, assessment, and management: emotional, behavioural, and cognitive. Eight studies used a cognitive approach for intervention; however, they used emotional and behavioural categories in their evaluation and outcomes.

This scoping review provides information regarding how the biopsychosocial approaches in pain are integrated into the treatment of persistent shoulder pain. We included the studies that added the psychosocial dimension to other interventions regarding managing shoulder disorders and did not compare the effect of integrating or non-integrating the psychological approach in regular interventions.

Most of the studies used cognitive approaches as their intervention. However, some of the interventions have emotional and behavioural components as well, such as CBT. Psychological approaches in pain management alongside the positive effects on pain reduction reduced pain catastrophizing, fear of movement, kinesiophobia, depression, anxiety, and worry about pain. Three studies used a behavioural approach in treatment, and all of them reported a positive effect on reducing pain catastrophizing (cognitive). Only one of the studies used one approach (either behavioural or cognitive) in its evaluation and treatment. The effect of this linking was a reduction of pain catastrophizing (cognitive), fear of movement belief, anxiety, depression and worry (emotional), and pain intensity.

However, matching intervention components with the definition of the biopsychosocial model of pain was somewhat difficult due to the overlap between components/aspects to be integrated into the assessment, treatment, and evaluation of patients with musculoskeletal pain. Two other reviews also reported similar results in which it was not possible to identify how the cognitive-behavioural and emotional components used in treatments and evaluations were operationalized [34, 35]. This result highlights the importance of future studies to know how to effectively *integrate* the “cognitive, behavioural, and emotional components” in the management of musculoskeletal pain with a biopsychosocial theoretical approach.

This scoping review showed that not all treatments improved pain intensity. There is debate about whether reducing pain intensity should be a primary goal in persistent pain versus increasing activity/function while maintaining no worsening of pain intensity. This was illustrated in a trial of graded activity and pain education for widespread chronic pain, where improved psychological status and function were achieved without changes in pain intensity [36]. This is consistent with finding in this study where two interventions improved function, despite any benefit on pain intensity [25, 29].

Behavioural approaches by use of the GET may cause a reduction in kinesiophobia and pain catastrophizing and pain intensity in patients with shoulder pain [27, 29]. Graded exposure to exercise or GET directly affects movement-related pain memories, and the patients will experience exposure without danger, and this improves their participation in activities that are associated with pain based on their experience or beliefs. Graded exposure to activities can result in positive feelings and improved self-efficacy [37]. Progressive activities are an experiential way of influencing fear-avoidance beliefs as patients experience success. This can negate previously held maladaptive beliefs [38]. This method has been widely used in different persistent and chronic MSK pain [39–41]. However, there are some studies that have indicated that behavioural therapy does not add any benefit to routine exercises in pain reduction in patients with low back pain [42] or cancer [43].

Three studies [24, 26, 27] used CBT as their intervention approaches pain and pain-related psychological factors. The results of those studies showed that CBT improved psychological health, fear-avoidance, pain, and pain catastrophizing. Despite the small number of studies with different approaches, there are very preliminary indications that CBT may have a broad benefit as these three studies indicted positive effects on a larger array of outcomes than some of the psychological interventions. One of the CBT studies included in this review was an RCT, which confirms the potential of CBP for improving some outcomes for people with persistent pain [26]. Some patients in this study had just shoulder pain, and others had shoulder pain in combination with other sites. While this conclusion was not specific to shoulder pain due to their study sample composition, we cannot generalize the conclusion to be valid for patients with shoulder pain.

Two other studies used CBT in both arms of their trial. They compared the effectiveness of CBT with web base CBT [27] or matching the treatment to patients' profile [24]. Their results indicated improvement in pain and pain-related psychological factors in both arms.

It can be difficult to distinguish different treatments in this review, as there can be considerable overlap in approaches. For example, GET, exercises with a behavioural approach, Web-BCPA, Coping Strategies, Psychological Flexibility, and V.R. address at least one of the CBT modules. While graded activity seems to be more physically based, it is often executed to challenge negative cognitions or use as the platform to discuss negative cognitions or expectations. It is meant to build self-efficacy and resilience while the emphasis is on physical or functional accomplishments. Conversely, cognitive approaches that rely more on discussion ultimately require patients to apply principles or challenge beliefs in their daily activities. Thus, separating these approaches ignores the common goals and strategies that they rely on.

The clinical application of our findings is to support the inclusion of psychological strategies in managing persistent shoulder pain. It suggests that both a physical approach with psychological overlay such as what happens in graded activity and a cognitive approach that leads to changes in physical activity/function may be beneficial. There is insufficient evidence to choose one specific approach over another. This means that clinicians and patients should be cautious when anyone proposes that they have the best approach to managing the psychological aspects of shoulder pain. This limits treatment algorithms and the translation of knowledge into the best practice. However, changing clinicians' beliefs about nociception, pain, and the effect of psychological factors is supported by this review and may start to improve practice and research.

### 4.2. Limitations

The purpose of a scoping review is to understand the state of the literature and how interventions are delivered within that literature, which presumably reflects current thinking on how to provide biopsychosocial treatment of chronic shoulder best. In this sense, we accomplished our aim and synthesized the literature to be informative for future design and intervention description. However, we were unable to conclude on choosing the best psychological intervention to be considered in pain reduction, and the flaws in the description of the rationale of the content of these interventions suggest that a systematic review might be preliminary.

Another limitation for our study was our sample selection. We had the problem of what to do with studies of mixed populations of MSK problems. We did not want to lose important data from studies where shoulder patients formed a substantial part of the sample, especially given the small number of eligible studies. Therefore, we chose a cut-off on what proportion from shoulder patients has to be included. We decided to keep one study in which 30% of the patients had shoulder or neck pain, and other patients in that study had general pain or back pain due to its design and sample size, matching the psychological profile with treatment since we thought it added value to our conceptual understanding. We acknowledged that patients with shoulder pain could have pain at other sites and patients who were labelled as widespread pain may have shoulder pain. Not all studies are clear on this. So, we are uncertain about the percentage of patients suffering specifically from shoulder pain. Thus, we presented results as a descriptive synthesis in order to describe the diversity in evidence. However, we could not evaluate the superiority of the various interventions and the size of their treatment effects or the relative effectiveness of different types of psychological interventions on shoulder pain.

### 4.3. Conclusion

Limited studies integrated psychological interventions in their treatment approaches to reduce pain intensity in patients with persistent troubling shoulder pain. None of the studies thoroughly explained how the three pain-related psychological factors (cognition, emotion, and behavioural) were targeted in evaluation, treatment, and outcomes. The available evidence showed that integrating behavioural approaches in intervention could help in reducing pain catastrophizing and kinesiophobia and consequently pain intensity. Cognitive approaches had a positive association with reductions in the emotional aspect of pain. Limited evidence showed that matching the evaluation and intervention can improve the outcomes. There are limited studies that reported integrating psychological intervention in pain reduction, and it is not possible to report a systematic review on this topic.

This makes it difficult to discern the important aspects of treatment or to replicate treatment approaches. Efforts should be directed to building clear conceptual frameworks that link treatment targets and interventions commonly used in targeting psychosocial treatment, definitions, and appropriate psychological outcome measures so that future RCTs would provide a clear understanding of the best treatment algorithm for chronic shoulder pain.

## Figures and Tables

**Table 1:** Study characteristics.

Study	*N* (female)	Diagnosis	Intervention	Control	Study design	Outcome	Follow-up	Results
[28]	176 (102)	Persistent shoulder pain (3 months or more)	Behavioral and time-contingent graded exercise therapy program	Usual care	RCT	Performance of the level of daily activities, perceived recovery, shoulder pain, generic health-related quality of life, catastrophizing, coping with pain, kinesophobia, and fear-avoidance beliefs	12 W	Graded exercise therapy with focus on behavioral changes was more associated with restoring the performance of daily activities (mean difference = 7.5, *p* = 0.04) and pain and catastrophizing thoughts (mean difference = 1.7, effect size = 0.07) compared to usual care. Pain reduction was significantly associated with reduction of depression scores (effect size = 0.1, *B* = 7.6)
[27]	109 (84)	Persistent pain in the back, neck, or shoulder, or generalized pain	The Web Behavior Change Program for Activity (Web-BCPA)	Multimodal rehabilitation Synchronized treatments based on a biopsychosocial perspective of pain and with the patient in focus	RCT	Pain intensity, self-efficacy, copying, adherence, feasibility, and treatment satisfaction	4 M, 12 M	Adding a self-guided Web-based intervention with a focus on behavioral change to MMR reduced catastrophic thinking (effect size = 0.61) at 12 months. However, it had no effect on pain and self-efficacy improvement. In both groups, pain was reduced and self-efficacy and coping improved
[24]	105 (76)	Workers at risk for pain-related disability	Matching treatment with psychological profile (activity training, graded exposure, and cognitive behavioral treatment)	Unmatched treatment	RCT	Perceived disability, sick leave, self-rated health status, fear and voidance, pain intensity, pain catastrophizing, depressive symptoms, anxiety, worry, and health care consumption	9 M	All participants experienced improvement in perceived disability, sick leave, fear and avoidance, pain catastrophizing, and distress (effect sizes *d* ranging between 0.23 and 0.66), but there was no benefit to matching. Pain intensity was improved in both groups with medium effect size. There was no standard treatment control to determine if these improvements occur without specific psychological intervention
[25]	37 (22)	Frozen shoulder	Emotional freedom technique (EFT) (exposure therapy and cognitive therapy, with acupoint stimulation)	EFT with diaphragmatic breathing and wait list	RCT	ROM, pain, and psychological conditions (anxiety and stress)	1 M	Strangely, the authors only analyzed what happened within each group, and there are no cross-group comparisons. The groups look comparable at enrollment with very slightly lower means in pain and psychological measures in the acupressure group compared to diaphragmatic breathing and wait list at one month. These differences are unlikely to be statistically significant and are small and clinically insignificant, suggesting no measurable effect of a single treatment. There was no change in shoulder motion
[29]	216 (126)	Persistent shoulder pain	Neck-specified exercise with a behavioral approach: (graded exercise, education)	Neck-specified exercise without a behavioral approach or prescription of physical activity	RCT	Pain disability, pain catastrophizing, anxiety, depression, and kinesophobia	0, 3, 6, 12, 24 M	Adding a behavioral approach to neck-specific training exercises decreased general pain limitations and pain catastrophizing more than neck-specific exercise alone (at least 50% (*p* < 0.01). This method of treatment decreased pain limitations and catastrophizing in the short term, and the patients sustained this over 2 years in comparison to neck-specific exercises alone. This method also decreased kinesophobia in the first year and anxiety over 2 years
[26]	469 (298)	Shoulder, back, and neck pain	Cognitive behavioral treatment in addition to physical treatment, cognitive behavioral modification, education, and examination of the work situation	Usual care	RCT	Return to work. Work conditions, life quality, physical activity and training, pain, subjective health, psychological changes, and practical performance	0, 4, and 12 M	Adding a cognitive behavioral therapy to physical therapy helps in decreeing pain. CBT in addition to physical treatment significantly decreased the pain intensity than use of physical treatment alone (*t*, 127) = 6.50, *p* < 0.001). The intervention had no affect on return to work of the patients. However, improvement in ergonomic behavior, work potential, life quality, and physical and psychological health achieved
[32]	112 (112)	Persistent neck, shoulder, upper back, lower back, pain	Individually adapted approach with a focus on physical exercise, mindfulness, and education on pain and behavior	Usual care	RCT	Fear avoidance	0, 10 W	Individually adapted physical-cognitive-mindfulness training in comparison to routine physiotherapy approaches significantly reduced work-related fear avoidance. This method could not significantly improve leisure time activity-related and fear-avoidance beliefs, by 10 weeks in comparison to the control group
[33]	2290 (1559)	Persistent pain after neck strain injuries	Pain coping strategies: active coping (attempts to engage the physical activity in spite of the pain) and passive coping (withdrawing from activities due to pain).	—	Prospective cohort	Global recovery	3, 6, and 9 M	Low level of passive coping strategy in the people with depressive symptoms causes recovery four times more quickly than those with depressive symptoms and high levels of passive coping. Active coping strategies showed no independent association with recovery
[18]	114 (73)	Persistent shoulder pain	Psychological flexibility (promotion of acceptance, mindfulness, values-based action, and cognitive diffusion) and traditional pain management	—	Prospective cohort	Eight measures of functioning: pain, pain-related anxiety, depression, physical disability, psychological disability, walking distance, and sit to stand	0,3 M	Pain-related outcome changes in from pre- to posttreatment are more related to psychological flexibility than traditional pain managements
[30]	6 (6)	Persistent shoulder or arm pain after breast cancer surgery	BrightArm Duo therapy (a robotic platform with various 3D games that address memory and cognition along with movement) for pain limitations after breast cancer surgery	—	Prospective cohort	Pain and disability	8 W	Eight-week treatment with this program led to twenty percent pain reduction, with a significant 8.3-point reduction in depression severity (*p* = 0.04) and increase range of motion; as there was no control group, it cannot be concluded that the improvement is merely due to virtual rehabilitation.

**Table 2:** The psychological construct measured, intervention, and outcome measures used in the articles.

Study	Primary construct measured (outcome measures)	Secondary construct measured (outcomes measures)	Effect on primary outcome	Effect on secondary outcomes	Effect on pain
[28]	Performance of the level of daily activities (SDQ)	Perceived recovery (eight-point ordinal scale) Shoulder pain (SPS) Quality of life (EuroQol-5D) Catastrophizing (PCCL) Coping with pain (PCCL) Kinesophobia (TSK-DV) Fear-avoidance beliefs (FABQ) Internal Locus of Control (PCCL) External Locus of Control (PCCL)	Mean difference = 7.5, *p* = 0.04	Catastrophizing thoughts (mean difference = 1.7, effect size = 0.07)	Effect size = 0.1, *B* = 7.6
[27]	Pain (VAS)	Self-efficacy (ASES) (GSE) Coping (CSQ) Adherence (minutes spent in each module) Feasibility and treatment satisfaction (A set of 8 items made by the authors)	No significant improvement	Catastrophic thinking (12 months: effect size = 0.61, *p* = 0.003)	NA
[24]	Perceived disability (QBPDS) Sick leave (days off work)	Health status (EQ-5D) Fear and avoidance (TSK) Fear-avoidance beliefs (FABQ) Pain VAS Pain catastrophizing (PCS) Depressive symptoms and anxiety (HADS) Worry 4-item global worry rating Health care consumption 4 items asking for number of visits to clinicians	Perceived disability (*d* = 0.40)	Health status: *F* = 3.76 Fear and avoidance: *d* = 0.66 Pain catastrophizing: *d* = 0.35, *d* = 0.48 Depressive symptoms *d* = 0.34, *d* Anxiety *d* = 0.51 Worry *d* = 0.41	Posttreatment effect size = 0.51 Follow-up effect size = 0.23
[25]	Range of motion: (goniometry)	Psychological symptoms (Symptom Assessment-45) Psychological distress anxiety, depression, obsessive-compulsive behavior, somatization, phobic anxiety, hostility, interpersonal sensitivity, paranoid ideation, and psychoticism Pain VAS	No benefit	Anxiety, pain, and depression	No effect
[29]	Pain disability: (PDI)	Pain catastrophizing: (PCS) Anxiety and depression (HADS) Kinesophobia (TSK)	Pain disability improved in short term, and disability was maintained over time (6, 12, and 24 months; *F* = 6.3, *p* < 0.01)	Pain catastrophizing decreased from baseline to 12 m for NSE (*F* = 6.9) group, and from baseline to 24 m for NSEB group (*F* = 6.3) Kinesophobia was improved from baseline to 12 m in NSE group (*F* = 7.6, *p* < 0.01) No significant effect on anxiety and depression	NA
[26]	Return to work: Days off work	Pain intensity (VAS) Daily activities (ADS) Subjective health (UHI) Health locus of control (MHLC) Anxiety (STAI I-II) Psychological distress (HSCL) Personality (EPI)	No effect	Improved psychological health (risk ratio: 1.61)	No effect
[32]	Fear avoidance (FABQ)	Pain intensity VAS	Reduced work-related fear avoidance: effect size = 0.3		Reduced (no data provided)
[33]	Global recovery self-reported global recovery	Pain intensity VAS	Using passive coping strategies slower recovery rate of 37%	NA	Pain reduced
[18]	Physical and psychosocial disability sickness impact profile	Pain intensity (VAS) Depression (British Columbia Major Depression Inventory) Pain-related anxiety and avoidance (Pain Anxiety Symptoms Scale-20)	Chronic pain treatment outcomes (−0.41 < *r* < −0.19) except for depression (-0.04)		Pain reduction was more associated with psychological flexibility (*r* = −0.19)
[30]	Pain intensity (NRS)	Depression (BDI-II)	20% pain reduction	Reduction in depression level (*p* = 0.04)	20% pain reduction

SDQ: Shoulder Disability Questionnaire; SPS: Shoulder Pain Score; PCCL: Pain Coping and Cognition List; TSK: Tampa Scale for Kinesophobia; FABQ: Fear Avoidance Belief Questioner; PCS: Pain Catastrophizing Scale; ASES: Arthritis Self-Efficacy Scale; GSE: General Self-Efficacy Scale; CSQ: Coping Strategies Questionnaire; VAS: Visual Analogue Scale; QBPDS: Quebec Back Pain Disability Scale; HADS: Hospital Anxiety and Depression Scale; EQ-5D: Euro Qol questionnaire; PDI: pain disability index; ADS: Activity Discomfort Scale; UHI: Ursin's Health Inventory; MHLC: Multidimensional Health Locus of Control; STAI I-II: Spielberg State Trait Anxiety Scale; HSCL: Hopkins Symptom Checklist; EPI: Eysenck Personality Inventory; BDI II: Beck Depression Inventory Second Edition.
